# Assessment of Roll-Over Test in Preeclamptic and Healthy Pregnant Women Using Arterial Stiffness Measurements—Prospective Case–Control Study

**DOI:** 10.3390/jcm14092897

**Published:** 2025-04-23

**Authors:** Szilárd Szatmári, Dániel T. Nagy, Bence Kozma, Dénes Páll, Zoltán Szabó, Béla Fülesdi, Petronella Hupuczi

**Affiliations:** 1 Department of Anesthesiology and Intensive Care, University of Debrecen Faculty of Medicine, 4032 Debrecen, Hungary; szatmari.szilard@med.unideb.hu (S.S.); nagy.daniel.tamas@gmail.com (D.T.N.); 2 Obstetrics and Gynecology, University of Debrecen, 4032 Debrecen, Hungary; bence.kozma@med.unideb.hu; 3 Faculty of Medicine, Internal Medicine University of Debrecen, 4032 Debrecen, Hungary; pall.denes@gmail.com; 4 Department of Medical Clinical Pharmacology, University of Debrecen, 4032 Debrecen, Hungary; 5 Department of Emergency Medicine, Faculty of Medicine, University of Debrecen, 4032 Debrecen, Hungary; szaboz.med@gmail.com; 6 Neuroscience Doctoral School, University of Debrecen, 4032 Debrecen, Hungary; hupuczip@gmail.com; 7 Maternity Hospital Budapest, 1126 Budapest, Hungary

**Keywords:** preeclampsia, applanation tonometry, arterial stiffness measurements, roll-over test

## Abstract

**Background:** The early recognition of systemic hemodynamic changes resulting from uteroplacental circulation disturbance in preeclampsia (PE) is of great importance for its appropriate treatment and prevention. The aim of the present study was to assess the hemodynamic changes during a roll-over test in healthy normotensive and preeclamptic pregnant women using applanation tonometry. **Patients and methods:** Healthy pregnant and PE women in their third trimester were studied. First, applanation tonometry was performed in a resting state on the right radial artery of each subject. In the second phase, the measurements were repeated in the left-lateral position and 5 min after turning each patient into a supine position (roll-over test = ROT). The systolic and diastolic central and peripheral blood pressures, pulse pressures, and augmentation index (AIx_75_) values were registered for all phases. **Results:** A total of 21 PE and 14 healthy pregnant women entered this study. At rest, the PE patients had higher systolic, diastolic, and mean blood pressures; the preeclamptic patients had higher peripheral and central blood pressure and pulse pressure values compared to the healthy controls. A statistically significant difference was found between the augmentation index (AIX-75) values for the preeclamptic and healthy pregnant women (healthy pregnant: 9.0 ± 2.4 vs. preeclamptic: 18.9 ± 6.0; *p* = 0.019). During the ROT, no significant differences could be detected in the applanation tonometry parameters within the groups. The differences between the PE and healthy pregnant women continued to exist in the left-lateral and supine positions during the roll-over test. **Conclusions:** This is the first study combining a roll-over test and arterial stiffness measurements in healthy pregnant females and in those with PE. Although we can confirm that arterial stiffness measurements can be used to detect hemodynamic changes in pregnant women with PE, combining it with a roll-over test is unsuitable for improving the method’s sensitivity.

## 1. Introduction

Preeclampsia remains a significant challenge in obstetric care, as it is one of the most common risk factors for adverse peripartum maternal and fetal outcomes worldwide [1]. Systemic hemodynamic changes are the characteristic features of the condition. In a normal pregnancy, the plasma volume increases by 50% and the cardiac output by 40–50%, accompanied by a 25–30% decrease in the peripheral resistance [2,3,4]. The primary purpose of this is to increase the uteroplacental blood flow to meet both the maternal and fetal metabolic demands. Based on previous studies, it is assumed that in pregnant women susceptible to preeclampsia, the adaptation of the circulatory system observed in a normal pregnancy is absent. One of the most widely accepted theories is that the primary cause of preeclampsia is an impaired invasion of trophoblasts into the maternal spiral arteries, resulting in placental hypoperfusion and hypoxia [5,6]. Placental hypoxia triggers inflammatory processes that disrupt the balance of the NO–endothelin system. As a consequence of the excess of vasoconstriction, an increase in blood pressure and target organ damage (e.g., brain, kidneys) may develop [7,8].

Although some of the data in the literature suggest that there are already signs of uteroplacental circulatory maladaptation in the first trimester of pregnancy [9], there is currently no method with sufficient sensitivity and specificity for the early prediction of preeclampsia. Combined models have achieved some degree of early prediction ability [10]; however, their sensitivity remains relatively low. A recently published prospective study suggested that arterial stiffness measurements may predict the later onset of preeclampsia earlier (as early as first trimester) and more sensitively than blood pressure measurements, uterine artery Doppler results, or angiogenic biomarkers [9].

Applanation tonometry is a promising method that allows for simple, non-invasive, and repeatable measurements of the central blood pressure, arterial wave reflection, pulse wave velocity, and arterial stiffness [11]. In women with PE, the pulse wave velocity and augmentation index (AIx) were both shown to be increased compared to those of the normotensive controls [12], and other studies have also confirmed the elevation of the AIx [13,14,15,16].

Another simple test method suitable for examining hemodynamic changes in preeclampsia is the roll-over test [16]. This test is based on the observation that turning from the left-lateral to the supine position results in an increase in blood pressure, both in healthy pregnant women and those with preeclampsia, but the magnitude of the blood pressure increase is higher in the preeclampsia group [17,18]. The discriminative power of the roll-over test alone for predicting preeclampsia is moderate [19], but it is not known whether, in combination with other methods, it may be a useful tool for assessing the hemodynamic differences between preeclamptic and healthy pregnant women. The aim of the present study was to assess the hemodynamic changes during a roll-over test in healthy normotensive and preeclamptic pregnant women in combination with applanation tonometry.

## 2. Methods

Healthy and preeclamptic patients of the Department of Obstetrics and Gynecology, University of Debrecen, were included. This study was approved by the Medical Ethics Committee of the University of Debrecen under registration number of DE RKEB/IKEB—7056-2025, with approval date of 7 January 2025. Informed consent for participation was obtained from all subjects involved in this study. Healthy pregnant women were outpatients with no previous or present signs of hypertension, diabetes mellitus, or significant renal disease in their history. The Department of Obstetrics and Gynecology, University of Debrecen, serves as a regional center for complicated pregnancies. Pregnant women with preeclampsia were selected by the obstetric outpatient department through regular checking of their blood pressure and proteinuria. Women who were considered high-risk were admitted to the department as inpatients. Standard criteria were used for selecting the preeclamptic patients: at least 20 weeks gestational age, de novo hypertension, blood pressure exceeding 140/90 mmHg on three different occasions, and proteinuria higher than 0.3 g/day.

SphygmoCor (AtCor Medical, Sydney, Australia) device was used for applanation tonometry. The sensor was placed on the patient’s skin over the radial artery. In the present study, parameters were recorded at the left radial artery because the left-lateral position during roll-over test allowed for better and more stable accessibility of the left arm. During the measurements, the registration and the analysis of the reflected wave by the sensor was possible. Based on previous studies central aortic pressure is sensitive predictor of future cardiovascular risk whereas augmentation index is an indicator of the arterial stiffness [11,20]. Systolic and diastolic aortic pressure, central aortic pulse pressure, peripheral pulse pressure, augmentation pressure, and augmentation index normalized on actual heart rate (Alx75HR) were registered at predetermined times during this study. All measurements were performed by two experienced operators (D.P. and Z.S.) who were unaware of the grouping status of the patients (preeclamptic or not). Applanation tonometry measurements were performed on 3 occasions: in resting state in the supine position; after 5 min resting period, in the left-lateral position during the roll-over test; and in the supine position during the roll-over test. **Statistical analysis:** SigmaPlot for Windows version 11 (Systat Software Inc., Palo Alto, CA, USA) program was used for statistical analysis. Means and standard deviations are presented for all values. Statistical analysis was started using normality tests before performing any comparisons. Normally distributed data between the groups were compared using the appropriate *t*-tests, while for comparison of data showing non-normal distributions, Mann–Whitney rank-sum test was used. Within-group comparisons were performed using paired *t*-tests. Non-parametric values of medical history were compared by chi^2^ tests. A *p* < 0.05 was accepted as the level of statistical significance.

## 3. Results

Twenty-one preeclamptic and fourteen healthy pregnant women entered this study. The most important data about their previous general medical and obstetric history are summarized in Table 1. In the preeclamptic group, the number of previous preeclamptic episodes were more frequent. Urine microalbumin levels were higher in the preeclamptic patients. Otherwise, there were no significant differences between the gestational histories of the actual pregnancies.

### 3.1. Comparison of Applanation Tonometry Results Between Healthy Pregnant and Preeclamptic Patients at Rest

**Figure 1:**
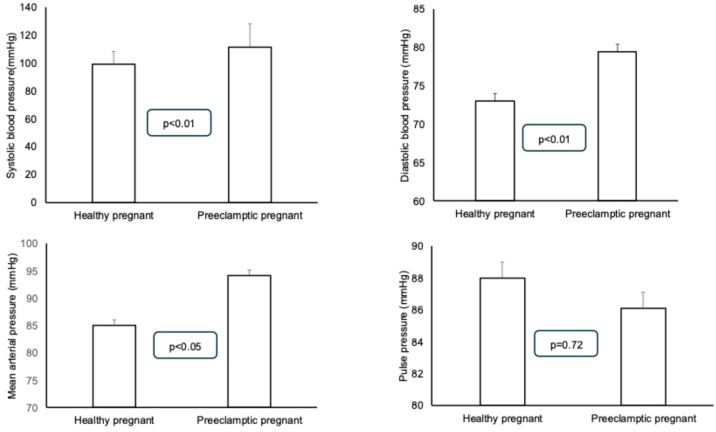
Comparison of systolic, diastolic, mean, and pulse pressure values of preeclamptic and healthy pregnant patients. Means ± standard deviations are presented.

**Figure 2:**
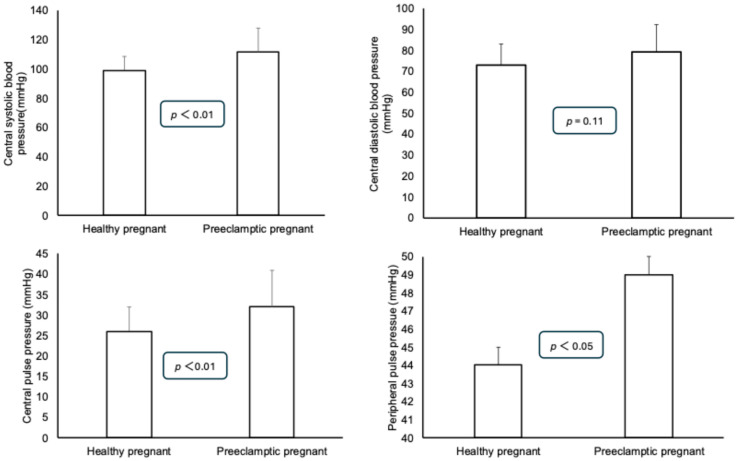
Comparison of central systolic, diastolic, and pulse pressure values for preeclamptic and healthy pregnant patients. Means ± standard deviations are presented.

Statistically significant differences could be detected between the systolic, diastolic, and mean blood pressures in a resting state: the preeclamptic patients had higher blood pressure values. No differences could be detected between the pulse pressures of the two groups (Figure 1). Among the parameters derived from the measurement curve, the central systolic and pulse pressures were significantly higher in the preeclamptic patients. However, no difference was found in the central diastolic blood pressure between the two groups. Similar to the central pulse pressure, the peripheral pulse pressure was also found to be higher in the preeclamptic patients (Figure 2). The augmented pressure was also higher in the preeclamptic pregnancies (5.9 ± 2.8 mmHg) than in the healthy pregnant women (1.0 ± 0.6 mmHg, *p* < 0.01). A statistically significant difference was found between the augmentation index (AIX-75) values of the preeclamptic and healthy pregnant women (healthy pregnant: 9.0 ± 2.4 vs. preeclamptic: 18.9 ± 6.0; *p* = 0.019).

### 3.2. Hemodynamic Parameters During Roll-Over Test

The results of the applanation tonometry measurements for the healthy and preeclamptic pregnant women are summarized in Table 2 and Table 3. There were no statistically significant changes in any of the hemodynamic parameters during the roll-over test within the groups. In the preeclamptic patients, the central and peripheral pressures and augmentation index values were slightly higher after turning from the left-lateral to supine position, but these differences did not reach the level of statistical significance.

The differences between the preeclamptic and healthy pregnant women that were observed during the resting state continued to exist in the left-lateral and supine positions during the roll-over test.

## 4. Discussion

In the present study, we used a combination of two methods for assessing the hemodynamic parameters of preeclamptic and healthy pregnant women. This is the first investigation that combines non-invasive applanation tonometry with the roll-over test. Although both methods have been used before as screening tools for preeclampsia, based on the results of the present study, the roll-over test in combination with an assessment of arterial stiffness does not add further information for a better understanding of the pathophysiological changes in preeclampsia.

The roll-over test (ROT) is based on observations that a change in posture results in changes to the systemic blood pressure of pregnant women in the third trimester [21]. It has also been shown, through the use of the roll-over test in preeclampsia, that in pregnant women susceptible to preeclampsia, turning from the left-lateral to the supine position results in an increase in the diastolic blood pressure of more than 20 mmHg. There are conflicting results available on its usefulness, ranging from an 88% positive predictive value [22] to a 20% positive predictive value [23]. In a follow-up study by Andersen, repeated blood pressure measurements during roll-over tests enabled the prediction of hypertension in roughly half of the cases [24]. In a more recent comparative study, a roll-over test alone had a sensitivity of 47%, a specificity of 90%, a PPV of 35.7, and an NPV of 94%, whereas, in combination with elevated uric acid levels and BMI, the PPV was as high as 100% [25]. The systemic blood flow changes observed during roll-over tests have also been used for testing static cerebral autoregulation in preeclamptic patients of different severity [26,27]. These recent results led us to the hypothesis that the combination of arterial stiffness measurements with a roll-over test may enhance the accuracy of the diagnosis.

Arterial stiffness measurements in pregnant women with preeclampsia have consistently demonstrated increased stiffness. Furthermore, arterial stiffness measurements have enabled the differentiation between preeclampsia and gestational hypertension [28]. In addition to the fact that arterial stiffness parameters are increased in preeclamptic pregnancies in the third trimester, similar differences can be detected as early as the second trimester [29,30]. In the early course of a pregnancy, an increase in the arterial stiffness is a more sensitive indicator of susceptibility to preeclampsia than blood pressure measurements, uterine artery Doppler indices, and angiogenic biomarkers [9]. The importance of arterial stiffness testing is also underlined by the long-term observation that mothers with increased arterial stiffness during pregnancy have a higher chance of giving birth to small-for-gestational-age children [31], and that the incidence of cardiovascular disorders in these mothers is increased in their later lives [32].

With the use of applanation tonometry to assess arterial stiffness, increases in the central and peripheral systolic and diastolic pressures, as well as pulse pressures, have been documented; in addition, most studies have described increases in the pulse wave velocity (PWV) and the augmentation index, which is considered the main marker of arterial stiffness. In their study, Robb and associates demonstrated an increase both in the PWV and AIx [12]. Others have reported increases in the augmentation index [13,14,15]. These findings are in accordance with our findings, as we demonstrate that the central and peripheral blood pressures, as well as the pulse pressures, are increased in preeclamptic patients. The augmented pressures and augmentation index are also found to be higher in the preeclamptic pregnants at rest compared to the healthy normotensive pregnants.

Combining a roll-over test and applanation tonometry for the assessment of the hemodynamic changes in preeclampsia has not been tested before by other investigators. Although the results of the arterial stiffness measurements were able to differentiate between the preeclamptic and healthy pregnant women in a resting state, during the roll-over test no further differences could be detected. The roll-over test was originally meant as an easy-to-perform screening method for the detection of preeclampsia. As described above, the sensitivity and the positive predictive value of the test have shown a wide range in different studies [22,23,24,25], and are very much dependent on the severity of the actual symptoms and the effectiveness of the treatment. In the present study, mainly preeclamptic patients with medium severity were included, and these patients were undergoing antihypertensive treatment. These two factors may explain why only discrete elevations in blood pressures were detected during turning from the left-lateral to supine position. As a consequence, combining a roll-over test with applanation tonometry methods did not yield further additional information about the hemodynamic pathophysiological changes in preeclamptic patients.

We have to mention the limitations of our study. First, we did not record the pulse wave velocities (PWVs) during applanation tonometry. However, as described above, increases in the PWVs have been somewhat inconsistent in previous studies. Hence, most of the investigations have focused on the assessment of the augmentation index, which has been demonstrated to be the most sensitive parameter for the diagnosis of altered arterial stiffness. Therefore, we also chose this parameter. Second, we may be criticized for the relatively low number of subjects in the present study (14 healthy and 21 preeclamptic pregnant women). This study was considered a pilot investigation, in which we intended to test the administration of two non-invasive methods (applanation tonometry and roll-over test) in combination that are used in the diagnosis of preeclampsia.

In conclusion, our study confirms that arterial stiffness measurements can be used to detect hemodynamic changes in pregnant women with preeclampsia. Based on our study’s results, combining it with a roll-over test is unsuitable for improving the method’s sensitivity.

## Figures and Tables

**Table 1:** Family and individual medical and obstetric history of the participants.

	Healthy (*n* = 14)	Preeclamptic (*n* = 21)	chi^2^; *p*-Value
**Family history**			
**Hypertension**	5	17	chi^2^ = 7.4; *p* < 0.01
**Preeclampsia**	0	2	chi^2^ = 1.4; *p* = 0.23
**Intrauterine growth retardation**	0	0	chi^2^ = 0; *p* = 1
**Diabetes**	1	6	chi^2^ = 2.4; *p* = 0.12
**Individual patient data on previous history**			
**Hypertension**	2	5	chi^2^ = 0.47; *p* = 0.49
**Diabetes mellitus**	2	2	chi^2^ = 0.19; *p* = 0.66
**Kidney disorder**	1	3	chi^2^ = 0.43; *p* = 0.51
**Smoking**	0	0	chi^2^ = 0; *p* = 1
**Hypercholesterinemia**	1	0	chi^2^ = 1.54; *p* = 0.21
**Other (Leiden mutation, hyperthyreosis, hypothyreosis)**	3	2	chi^2^ = 0.97; *p* = 0.32
**Gestational history**			
**Previous pregnancies** **(number of pregnancies/patients)**	1 = 7 2 = 1 3 = 6	1 = 16 2 = 2 3 = 1 4 = 1 5 = 1	chi^2^ = 8.3; *p* = 0.07
**Previous deliveries** **(number of deliveries/patients)**	0 = 10 1 = 3 2 = 4 3 = 1	0 = 18 1 = 3	chi^2^ = 7.1; *p* = 0.07
**Spontaneous abortions** **(number of abortions/patients)**	0 = 9 1 = 5	0 = 20 1 = 2 2 = 5 3 = 1	chi^2^ = 7.7; *p* = 0.05
**Artificial abortions** **(number of abortions/patients)**	0 = 12 1 = 2 2 = 3	0 = 20 1 = 0 2 = 4 3 = 2	chi^2^ = 4.5; *p* = 0.21
**Preeclampsia**	1	12	chi^2^ = 8.9; *p* < 0.01
**Data on present pregnancy**			
**Age (years)**	29.5 ± 5.2	27.1 ± 5.7	*p* = 0.21
**BMI (kg/m^2^)**	25 ± 4.8	26 ± 5.5	*p* = 0.42
**Gestational weeks**	34.7 ± 3.5	35.2 ± 3.9	*p* = 0.82
**Urine albumin concentration**	0.057 ± 0.039	0.61 ± 0.85	*p* < 0.01
**Gestational weeks at birth**	38 ± 2.2	38 ± 1.98	*p* = 1

**Table 2:** Results of applanation tonometry measurements during roll-over test in healthy normotensive pregnant women. Means ± standard deviations are presented.

	Left-Lateral Position	Supine Position	*p*-Value
**Systolic blood pressure (mmHg)**	111.4 ± 12.4	112.4 ± 10.9	0.82
**Diastolic blood pressure (mmHg)**	71.0 ± 13.0	70.4 ± 9.6	0.89
**Pulse pressure (mmHg)**	81.3 ± 8.4	82 ± 10.5	0.83
**Central systolic pressure (mmHg)**	97.2 ± 11.3	100.8 ± 18.9	0.55
**Central diastolic pressure (mmHg)**	72.3 ± 13.2	71.4 ± 9.3	0.84
**Central pulse pressure (mmHg)**	25.0 ± 6.7	25.3 ± 6.5	0.88
**Peripheral pulse pressure (mmHg)**	39.7 ± 10.6	42.2 ± 12.2	0.56
**Mean arterial pressure (mmHg)**	83.3 ± 12.2	82.2 ± 8.6	0.85
**Augmented pressure (mmHg)**	2.28 ± 2.26	2.28 ± 3.9	1
**AIX 75**	11.6 ± 8.8	8.2 ± 10.7	0.37

**Table 3:** Results of applanation tonometry measurements during roll-over test in preeclamptic pregnant women. Means ± standard deviations are presented.

	Left-Lateral Position	Supine Position	*p*-Value
**Systolic blood pressure (mmHg)**	126.2 ± 17.2	127.2 ± 13.8	0.84
**Diastolic blood pressure (mmHg)**	73.1 ± 13.2	80.5 ± 10.8	0.07
**Pulse pressure (mmHg)**	83.0 ± 21.9	83.3 ± 15.1	0.92
**Central systolic pressure (mmHg)**	111.6 ± 15.4	112.1 ± 17.6	0.88
**Central diastolic pressure (mmHg)**	78.1 ± 13.1	81.5 ± 11.5	0.31
**Central pulse pressure (mmHg)**	33.5 ± 17.7	31.1 ± 9.2	0.4
**Peripheral pulse pressure (mmHg)**	50.2 ± 11.9	47.1 ± 9.15	0.35
**Mean arterial pressure (mmHg)**	93.2 ± 7.7	94.1 ± 12.5	0.75
**Augmented pressure (mmHg)**	5.35 ± 11.6	4.32 ± 6.4	0.63
**AIX 75**	16.8 ± 11.6	14.2 ± 12.2	0.51

## Data Availability

The data are available from the corresponding author upon reasonable request.
